# Feed Quality and Feeding Level Effects on Faecal Composition in East African Cattle Farming Systems

**DOI:** 10.3390/ani11020564

**Published:** 2021-02-22

**Authors:** Asep I. M. Ali, Shimels E. Wassie, Rainer Georg Joergensen, Daniel Korir, John P. Goopy, Klaus Butterbach-Bahl, Lutz Merbold, Uta Dickhoefer, Eva Schlecht

**Affiliations:** 1 Animal Husbandry in the Tropics and Subtropics, University of Kassel and University of Göttingen, 37213 Witzenhausen, Germany; asepali76@gmail.com (A.I.M.A.); eschlech@uni-kassel.de (E.S.); 2 Animal Nutrition and Rangeland Management in the Tropics and Subtropics, Institute of Agricultural Sciences in the Tropics, University of Hohenheim, 70599 Stuttgart, Germany; shimels_wassie@uni-hohenheim.de (S.E.W.); uta.dickhoefer@uni-hohenheim.de (U.D.); 3 Soil Biology and Plant Nutrition, University of Kassel, 37213 Witzenhausen, Germany; 4 Livestock, System and Environment, Mazingira Centre, International Livestock Research Institute (ILRI), Nairobi 00800, Kenya; d.Korir@cgiar.org (D.K.); j.goopy@cgiar.org (J.P.G.); klaus.butterbach-bahl@kit.edu (K.B.-B.); l.merbold@cgiar.org (L.M.); 5 Institute of Meteorology and Climate Research, Atmospheric Environmental Research (IMK-IFU), Karlsruhe Institute of Technology (KIT), 82467 Garmisch-Partenkirchen, Germany

**Keywords:** sub-Saharan Africa, Boran cattle, diet composition, fecal excretion, feed intake, manure quality, nutrient cycling

## Abstract

**Simple Summary:**

Sub-Saharan cattle are often exposed to a feed reduction caused by a seasonal lack of forage, which was investigated in the first experiment. The supplementation of roughage-based diets with sweet potato vine silage and urea molasses blocks is recommended to improve the growth of heifers, in particular, which was investigated in the second experiment. Across all data, the fungal C/bacterial C ratio was positively related to nitrogen and negatively to neutral detergent fiber concentrations in feces. This diet-induced shift in the fecal microbial community is relevant for the fertilizer quality of cattle faces after application to soil.

**Abstract:**

Effects of feeding levels below maintenance requirements of metabolizable energy (MER) and of feed supplementation on fecal nutrient and microbial C concentrations were evaluated. In experiment 1, Rhodes grass hay only was offered to Boran steers at 80%, 60%, and 40% of individual MER, while steers at 100% MER additionally received a concentrated mixture. This reduction in MER decreased N, increased fungal C but did not affect bacterial C concentrations in feces. In experiment 2, Holstein × Boran heifers were offered a poor-quality roughage diet without supplement, with sweet potato vine silage or with a urea-molasses block. These two supplements did not affect the fecal chemical composition or fungal C but increased bacterial C concentrations in feces. Across all data, the fungal C/bacterial C ratio was positively related to N and negatively to neutral detergent fiber concentrations in feces, indicating diet-induced shifts in the fecal microbial community.

## 1. Introduction

Inappropriate management of livestock dung can contribute to contamination of groundwater and surface water bodies with organic matter, nitrate, and organic phosphates [1]. Previous works [2,3] have shown that feed quality strongly affects the quality of manure and the rate and pattern of its decomposition and release of nutrients. In sub-Saharan Africa (SSA), cattle and other ruminants are seasonally exposed to a lack of forage, particularly at the end of the dry season and in drought years. In addition, in these periods, the available roughages are also of extremely poor quality, i.e., low in protein and energy concentrations but high in fiber fractions [4,5]. This results in low production of milk and weight loss [5,6,7].

Previous work has described the negative effects of underfeeding on nutrient digestibility [8] and animal physiology, nutrient and energy metabolism [9,10]. The drop in digestibility when ruminants are fed below their maintenance energy requirement (MER) may increase the amount and nutrient concentration of feces. For Holstein cows offered natural grassland hay, soybean meal, and barley, fecal nitrogen (N) loss increased from 350 to 450 g N kg^−1^ N intake when feeding level decreased from 110% to 65% MER [11]. An increase in fecal N loss from 410 to 830 g N kg^−1^ N intake was observed for sheep fed vetch-oats hay at 100% and 20% MER [12]. Fecal N loss was paralleled by a reduced N retention and an increased blood urea concentration [12], as well as a loss of endogenous N [8].

On the other hand, supplementing ruminants with protein-rich feedstuffs increased concentrations of fecal N, bacterial carbon (C), and total microbial C [13,14]. Feces from dairy cows on a high fiber and low crude protein (CP) diet had a higher C/N ratio than feces from cows on a low fiber and high CP diet, whereas fecal bacterial biomass was highest for the diet with the highest energy and protein content [13]. A difference in fecal composition and microbial biomass C/N ratio was also reported for Holstein cows on two diets differing in N concentration [14]. Higher fecal neutral detergent fiber (NDF) and hemicellulose concentrations were associated with an N deficient diet compared with an N balanced CP diet, whereas the amino sugar concentrations did not differ between these two treatments [14]. However, the amino sugars muramic acid (MurN) and fungal glucosamine (GlcN) were useful indicators for determining fecal bacterial and fungal biomass in animal feces of several other experiments [15,16,17].

When a diet high in fiber and low in CP is fed to cattle at below 100% MER levels, microbial growth in the hindgut may increase due to lowered rumen fermentation, resulting in an increased fecal N concentration from both bacterial mass and undigested feed N [18]. Similarly, GlcN and galactosamine (GalN) concentrations markedly varied when Boer goats consumed a mixture of ryegrass hay and concentrate that contained different levels of quebracho tannin and activated charcoal [16]. Tannin and charcoal additions lowered the digestibility of OM, CP, NDF, and acid detergent fiber (ADF) and, as a result, changed the fecal composition and increased fungal C concentration.

In the SSA context of smallholder cattle farming, little information has been provided on how diet composition and feeding level affect the nutrient concentration as well as the microbial composition of feces, even though this is a crucial aspect in nutrient balance studies [19]. Insights into the effects of common feeding situations in SSA, i.e., from sub-maintenance roughage feeding to quality supplementation of roughage diets, on the concentration of C, N, and microbial biomass in feces would be useful in reducing nutrient wastage by greenhouse gas emissions. Two experiments were carried out to investigate the following hypotheses: (1) A reduction in feed intake at levels below 100% MER will decrease fecal N and bacterial C but increase fecal ADF and fungal C. (2) A supplementation of a poor-quality roughage diet with quality supplements will increase fecal N and bacterial C but decrease fecal ADF and fungal C. (3) The fecal fungal C/bacterial C ratio gives sensitive information on diet-induced changes in feces composition, as fungi contribute a considerable percentage to the gut microbiome.

## 2. Materials and Methods

### 2.1. Experimental Feeding and Animals

A sub-maintenance feeding trial (experiment 1) and a supplementation trial (experiment 2) were conducted at the Mazingira Center of the International Livestock Research Institute, Nairobi, Kenya, during the period September to November 2015 (experiment 2) and July 2016 to January 2017 (experiment 1). The average minimum and maximum ambient air temperatures during experiment 1 were 18 °C and 20 °C, and relative air humidity ranged from an average minimum of 55% to an average maximum of 69%. During experiment 2, the average temperature and relative humidity ranged from 14 to 26 °C and from 17% to 93%, respectively. Animals in both trials were kept in individual pens (1.8 m × 2.8 m) in an open barn during three weeks of adaptation and in individual pens (1.1 m × 2.2 m) inside a closed barn during one week of measurements (see below). In both trials, drinking water was provided ad libitum.

The sub-maintenance trial was set up as a 4 × 4 Latin square, with twelve purebred Boran steers (African *Bos indicus*) of 183 ± 15.2 kg weight being assigned to four feeding levels, i.e., three animals per level, tested during the four periods (see below). Feeding levels were defined as 100%, 80%, 60%, and 40% of the animals’ individual MER of 0.74 MJ kg^−0.75^ live weight (LW), which is the MER for mature bulls [20]. Steers at 80% MER (MER80) as well as at MER60 and MER40 were only fed Rhodes grass hay, whereas steers at MER100 were given hay at 20 g kg^−1^ LW plus a concentrated mixture of cottonseed meal (CSM) and sugar cane molasses at 16 g concentrate per 100 g diet dry matter (DM). Molasses and CSM were mixed and fed in the morning, while hay was offered throughout the day until completely consumed. Supplementing steers at MER100 was necessary, as a diet based solely on the energy-poor hay (Table 1) would have exceeded the intake capacity of the animals at this feeding level. All trial steers always had access to a mineral lick block. The trial ran over four periods, each consisting of the following four parts: (1) three weeks of adaptation as a washout period, (2) one week of total fecal collection, (3) one week of respiration chamber measurements (not reported here [21]), and (4) two weeks of energy-rich refeeding. In this part, all animals received Rhodes grass hay ad libitum plus 2 kg CSM, 1 kg molasses and 100 g *Brachiaria* grass per day (all weights as fed). This feeding enabled animals of the sub-maintenance treatments (MER40, MER60, MER80) to regain LW before the subsequent adaptation part and minimized potential carryover effects of an altered energy metabolism. This experiment was approved by the Animal Care and Use Committee of ILRI (No. IACUC-RC2015-07) and the animals were under the constant observation of a veterinarian.

The supplementation trial was set up as a Youden square of two animals and two consecutive experimental periods. Six Holstein Friesian × Boran heifers (148 ± 4.6 kg) were allocated to three diets, namely a pure roughage (R) diet (61.4 g of wheat straw and 38.6 g of Rhodes grass hay per 100 g DM), a diet consisting of roughage and sweet potato vine silage (R + SPVS), and a roughage diet plus urea molasses blocks (R + UMB). Both experimental periods consisted of the following three parts: (1) three weeks of adaptation as a washout period, (2) one week of total fecal collection, and (3) one week of respiration chamber measurements (not reported here [21]). An energy-rich refeeding period to compensate for LW losses was not needed in this trial, as the heifers maintained their LW throughout the supplementation trial. The amount of roughage offered to each heifer was calculated from the weekly measured individual LW, allowing for a refusal of 5 to 10 g per 100 g offered roughage. The UMB was available ad libitum, while SPVS was offered at 2.5 g SPVS per 100 g LW (as fed), equivalent to 19 g SPVS per 100 g DM of R + SPVS. The roughages were chopped to 5–20 cm particle size and mixed daily, while the silage was prepared according to Lukuyu et al. [22], following the recommendation of Makkar et al. [23]. The UMB contained, in g 100 g^−1^ fresh matter (FM): water (5.0), magnesium sulfate (5.0), vegetable oil (1.0), sugarcane molasses (35.0), urea (10.0), sodium chloride (10.0), dicalcium phosphate (18.9), a trace mineral premix (Mn, Zn, Cu, Se; 0.1), cement (10.0) (Bamburi Cement, Nairobi, Kenya), and CSM (5.0). This experiment was approved by the Animal Care and Use Committee of ILRI (No. IACUC-RC2016-11) and these animals were also under the constant observation of a veterinarian.

### 2.2. Determination of Feed Intake and Fecal Excretion

After chopping and mixing, 100 g FM of offered roughage was sampled weekly and stored in a paper bag. Approximately 300 g FM of SPVS were collected when a new silage bag was opened; a sample of UMB (100 g FM) was collected in a plastic zipper bag at the moment of UMB preparation and stored at −20 °C. Molasses (70 g FM) and CSM (100 g FM) were sampled once per period. For each animal, refusals of roughage and SPVS were collected and weighed daily, while intake of UMB was quantified by calculating the weight difference of the block between two subsequent mornings during the whole measurement week. At the end of each measurement week, refusal of roughage was pooled and mixed before an aliquot of 100 g FM was sampled (CTG6H, Citizen Scales, New York, NY, USA). Quantification of refusals was done before morning feeding; in experiment 1, no refusals of the CSM-molasses mixture were left.

The total amount of fecal FM was collected per animal into a 10-L bucket from the clean floor at each time an animal defecated. Total fecal mass was weighed at 8:00 a.m. daily throughout the measurement week. The feces were thoroughly mixed, and an aliquot of 300 g FM was dried at 50 °C for 72 h and reweighed. For N analysis, another fecal aliquot of 60 g FM was collected and then stored at −20 °C. Dried samples were ground to pass a 1 mm mesh at the end of each experimental period and pooled per period, based on the daily amount of fecal excretion, homogenized, and kept for analysis. The frozen fecal samples were thawed, pooled, also based on the amount of daily excretion, mixed, and directly analyzed for total N. Mixing of feces with urine was excluded, as all animals wore funnels for urine collection [21,24].

To determine fecal amino sugars, about 50 g of fresh feces were sampled immediately after defecation at three different sampling times (12:00, 18:00, and 24:00 h) on days 2, 4, and 6 during each measurement week. If needed, the animals were manually stimulated at the anus to provoke defecation. After overnight storage in a zipper bag at −20 °C, each sample was transferred to a paper bag for vacuum freeze-drying at −55 °C and 0.4 mbar for 48 h in a Telstar LyoQuest-55 freeze-dryer.

### 2.3. Chemical Analysis of Samples

Ground and dried samples of feed, refusals and feces were analyzed for DM and organic matter (OM) concentrations (AOAC methods no 967.03 & 924.05 [25]), while NDF and ADF (VDLUFA methods no. 6.5.1 & 6.5.2 [26]) were determined in a Fiberec analyzer (Foss, Hamburg, Germany). The N concentration in pooled samples of frozen faeces was determined using the Kjeldahl procedure (AOAC method no. 988.05 [25]). MurN, GlcN, and GalN were analyzed in freeze-dried fecal samples of 400 mg [27]. They were hydrolyzed with 10 mL of 6 M HCl for 3 h at 115 °C and then filtered. From a 0.3 mL aliquot of the hydrolysate, HCl was removed using a vacuum rotary evaporator (Heidolph, Schwabach, Germany) at 40 °C. Then, the residue was dissolved in 1 mL bi-distilled water and centrifuged at 13,000 rev min^−1^ (Centrifuge 5910 R, Eppendorf) for 10 min, transferred to a plastic vial, sealed, and stored at −18 °C. Amino sugars were separated on a Phenomenex (Aschaffenburg, Germany) Hyperclone C18 column at 35 °C. The HPLC system consisted of a Dionex (Germering, Germany) P 580 gradient pump and a Dionex Ultimate WPS-3000TSL analytical autosampler with in-line split-loop injection and thermostat, using ortho-phthalaldehyde as reagent.

Fecal fungal C was estimated from GlcN and MurN concentrations as follows:

Fungal C = 9 × (mmol GlcN − 2 × mmol MurN)(1)

thereby assuming that the molar MurN to GlcN ratio in bacterial cells is 1 to 2 [28,29] and 9 is the conversion value of fungal GlcN to fungal C [30]. Bacterial C was calculated by multiplying the MurN concentration by 45, and microbial C was calculated as the sum of fungal C plus bacterial C [30].

### 2.4. Data Calculation and Statistical Analyses

Daily feed intake of the individual animal was calculated from the amount of daily feed offered and refused and its respective composition. The apparent digestibility of DM, OM, CP, NDF, and ADF was calculated by subtracting the amount of fecal excretion from the amount of ingested feed and dividing the difference by the ingested amount. The data on concentrations and amounts of fecal DM, OM, N, NDF, and ADF in the sub-maintenance trial (3 steers per each of the 4 treatments during 4 periods, resulting in 12 steers per treatment × 4 periods) and in the supplementation trial (2 heifers per each of the 3 treatments during 2 periods, resulting in 6 heifers per treatment × 2 periods), were analyzed for each experiment separately, using the following model:

y_ijk_ = µ + d_i_ + p_j_ + dp_ij_ + a_k_ + e_ijkl_ (2)

where y_ijk_ is the dependent variable for a particular ijk case, µ is the overall mean, d_i_ and p_j_ are the fixed effects of diet and period, respectively, dp_ij_ is the interaction of diet and period, a_k_ is the random effect of animal, and e_ijkl_ is the residual error.

For the data on amino sugar concentrations and microbial biomass, the data of experiment 1 (12 steers, 4 periods, 3 sampling hours, 3 sampling days) and experiment 2 (6 heifers, 2 periods, 3 sampling hours, 3 sampling days) were again analyzed separately using the following model:

y_ijkl_ = µ + d_i_ + p_j_ + dp_ij_ + s_k_ + ds_ik_ + a_l_ + e_ijklm_ (3)

where y_ijkl_ is the dependent variable for a particular ijkl case, µ is the overall mean, diet (d_i_) and period (p_j_) are fixed effects and their interaction (dp_ij_), the repeated measurements are accounted for via sampling day (s_k_) and its interaction with diet (ds_ik_), a_l_ is the random effect of animal and e_ijklm_ is the residual error. Tukey’s post hoc test was applied to detect significant differences (*p* ≤ 0.05) between diet, period, sampling day, and the interactions of the diet with period and sampling day.

In a first step, the data from the three sampling hours was included in the model to test for systematic diurnal variation in microbial markers. Because no significant differences in the concentrations of amino sugars and microbial C were observed between the different sampling hours within a day in both experiments (Table S1), only one data point per sampling day (the 12:00 h sample) was considered in the final analysis (Equations (2) and (3)). Statistical analyses were done using R v3.4.3 [31]. For ANOVA, the *lme* function was used, which allows the incorporation of fixed effect terms along with random effect terms into the linear model. In the repeated measurement models, a covariance structure with compound symmetry was fitted by using the *gls* function, thereby assuming that correlation between two measurements is equal across all experimental periods. For Pearson’s correlation, the *cor* function was used. All results are presented as arithmetic treatment means and standard error of the means (SEM).

## 3. Results

### 3.1. Feed Intake and Digestibility

In experiment 1, feed intake of OM, CP, NDF, and ADF all decreased (*p* < 0.05) with each decrease in MER level fed to the Boran steers (Table 2). The concentrate mixture given at MER100 (Table 1) markedly increased the CP digestibility but reduced the NDF and ADF digestibility to that of the MER40 level (Table 2). NDF and ADF digestibility increased in the order MER40 < MER60 < MER80, but the differences between these three MER levels were not always significant. Feed intake and diet digestibility showed significant variation between the four feeding periods for all fractions analyzed but no clear temporal trends. In experiment 2, feed intake and digestibility of OM, CP, NDF, and ADF were not affected by sweet potato vine silage (SPVS) and urea-molasses block (UMB) supplementation to the diet of Holstein × Boran heifers (Table 3). Feed intake and digestibility also did not differ between the two feeding periods. A significant diet × period interaction was only observed for the OM digestibility.

### 3.2. Feces Quality and Quantity

In experiment 1, fecal OM was significantly lowest at MER80 (Table 2), whereas fecal N was highest at MER100. Fecal NDF did not differ between the MER levels. ADF of MER80 was significantly less than that of MER60 and MER40, whereas ADF of MER100 did not differ from the other three MER levels. Fecal composition showed significant variation between the four feeding periods for all fractions analyzed, but again no clear temporal trends. In experiment 2, a difference in fecal composition was only observed for OM (Table 3), with similar concentrations for diets R + SPVS and R and the least for R + UMB. Fecal composition showed no significant variation between the two feeding periods for any fraction analyzed.

In experiment 1, the fecal excretion rates of OM, N, NDF, and ADF by Boran steers, standardized to 100 kg LW, significantly decreased by approximately 50% from MER100 to MER40 (Table 4), without significant differences between MER80 and MER60. In experiment 2, supplementation to the diet of Holstein × Boran heifers did not affect fecal excretion rates of the four fractions analyzed, which varied around 860 g OM, 17 g N, 700 g NDF, and 510 g ADF 100 kg^−1^ LW d^−1^.

### 3.3. Microbial Composition of Feces

In experiment 1, the diet of Boran steers did not affect fecal MurN and, thus, bacterial C concentrations (Table 2). GalN was significantly highest at MER80, whereas GlcN, fungal C, and microbial C were highest at MER60, but the difference to MER40 was not significant for GlcN and microbial C. The fungal C/bacterial C ratio varied around a mean of 0.49, i.e., fungi contributed 33% to microbial C. This ratio was lowest at MER100, but the difference to MER40 was not significant. All fecal microbial properties showed significant variation between the four feeding periods but no clear temporal trends, leading to numerous diet × period interactions.

In experiment 2, the supplementation to the diet of Holstein × Boran heifers increased the fecal concentrations of MurN, bacterial C, GalN, and microbial, but not those of GlcN and fungal C in comparison with pure roughage (Table 3). These changes reduced the fungal C/bacterial C ratio, especially in the diet with SPVS supplementation, which was significantly lower than that with UMB supplementation. The fungal C/bacterial C ratio varied around a mean of 0.77, i.e., fungi contributed 44% to microbial C. Concentrations of GalN, GlcN, and fungal C were higher in feeding period 2, leading partly to diet × period interactions.

### 3.4. Influence of Feed Intake and Diet Quality on Fecal Composition

Across the two experiments, fecal N significantly increased with increasing feed intake of CP, NDF, and ADF as well as with increased CP digestibility (Table 5) but significantly decreased with increased NDF and ADF digestibility. Fecal NDF and ADF decreased both with increasing feed intake of CP, NDF, and ADF, but only fecal NDF concentration decreased with increased CP digestibility.

Fecal GalN significantly decreased with increasing feed intake of CP and ADF (Table 6) as well as with increased CP digestibility and fecal N concentration. Fecal GalN showed positive correlations with NDF and ADF digestibility as well as with fecal NDF concentration. Fungal C was positively correlated only with the fecal N concentration. In contrast, bacterial C was negatively correlated with feed CP intake and fecal N concentration but positively with NDF digestibility and fecal NDF concentration. The fungal C/bacterial C ratio increased with increasing feed N intake and fecal N concentration but decreased with increasing NDF digestibility and ADF digestibility as well as with increasing fecal NDF concentration.

## 4. Discussion

### 4.1. Effects of Feed Intake and Diet Digestibility on Fecal Chemical Composition

The chemical composition of Boran steer feces was closely related to the MER level-specific feed intake of CP, NDF, and ADF, whereas the diet digestibility of these three fractions mainly affected fecal N concentration. The lower fecal OM concentration at MER80 than at MER60 and MER40 is in line with a higher fecal OM content of an N deficient diet [14] when considering the pure hay diets only. The higher fecal N loss as feed intake decreased, combined with negative energy and N balances of animals subjected to MER60 and MER40, suggest that supplemental feeding should be considered in this context [32]. In contrast to the MER level, the supplementation of the diet with SPVS and UMB to the Holstein × Boran heifers had no effects on feed intake, diet digestibility, or fecal chemical composition.

Across both experiments, the fecal N concentrations were similar to the 9 g N kg^−1^ DM reported for Friesian × Ayrshire steers in Kenya, purely fed with barley straw, and to the 11–17 g N kg^−1^ DM when this diet was supplemented with tree legumes and poultry manure [33]. However, fecal N concentrations were lower than those for forage alone and supplemented cattle diets in temperate regions of 17–38 g N kg^−1^ DM [13,14,34]. Increased feed intake of CP and CP digestibility resulted in increased fecal N concentrations, as observed by others [24,35]. Increased feed intake of CP and CP digestibility were negatively related to fecal NDF concentrations, as previously observed [14,36], whereas the similar strong relationships to ADF are only congruent with the data of Zhu et al. [36]. The negative effects of increased NDF and ADF intake on fecal NDF and ADF concentrations indicate complex interactions of these two fiber fractions during microbial decay in the rumen and lower gastrointestinal tract [17].

### 4.2. Effects of Diet Quality and Feed Intake on Fecal Microbial Composition

Across both experiments, the average concentrations of MurN and GalN, as well as that of microbial C in the feces of Boran steers and Holstein × Boran heifers, were in the range reported for Holstein heifers and cows in Germany [3,14,15,17]. In contrast, the average fungal GlcN concentration was slightly higher, which might be explained by the lower CP and higher NDF concentrations of the diets in the current two experiments. The fungal C/bacterial C ratios were lower in the feces from the MER100 diet of the Boran steers, which agrees with previous findings that higher feed intake N and lower feed intake NDF enhances fecal microbial biomass [3,13,14,15]. The lower fungal C/bacterial C ratios in the diets with SPVS and UMB supplementation to the Holstein × Boran heifers can be less easily explained with the current knowledge, as these two treatments did not affect feed intake of CP and NDF and also not the digestibility of these two fractions. However, sweet potato vine silage and urea molasses blocks both contain easily available organic matter, obviously supporting rumen and hindgut bacteria of Holstein × Boran heifers.

The lower CP and ADF digestibility at MER60 and MER40 than at MER100 and MER80 might have enhanced the supply of undigested feed N as well as NDF and ADF fractions to the lower gastrointestinal tract, stimulating fungal growth in the hindgut. A shift in the fecal microbial community towards fungi was reported after the addition of quebracho tannin to the diet, which reduced its digestibility [16]. This increase in the fungal C/bacterial C ratio was ascribed to a lowered N availability in rumen and hindgut due to the complexation of feed protein by tannins [16].

In the present study, the higher fungal C concentration at MER60, where feed intake of NDF and ADF were higher and intake of CP was lower than in the other diets, agree with previous findings [3,14,15,17]. However, the generally positive correlation of CP intake with the fungal C/bacterial C ratio and the negative correlation of CP intake with MurN across all data seem to contradict these results. One possibility might be a shift in bacterial community structure towards Gram-negative bacteria at MER60 and MER40 [17], which have a 70% lower MurN concentration than Gram-positive bacteria [29,30]. Another possibility might be a decrease in fungal cell volume, which results in overestimates of fungal C [30]. However, a negative relationship between fecal NDF and the fungal C/bacterial C ratio could simply mean that the decay of NDF in the hindgut was retarded due to the lower contribution of fungal biomass to the fecal microbiome. Similarly, the increased accumulation of hardly decomposable heterocyclic organic N components, such as purines and pyrimidines [37], during microbial decay processes in the hindgut might have reduced the contribution of bacterial biomass to the fecal microbiome. This may explain in part the regularly observed low N mineralization rates of cattle feces after application to soil [3].

Moreover, a higher ratio of fecal GlcN to fecal N was observed in pigs on a diet of maize starch, sucrose, and glucose (protein-free diet) than in pigs fed either a diet of wheat bran or a mixture of barley and wheat [38]. Therefore, further research is needed to compare the presently used methods of amino sugar quantification in animal feces with alternative methods of microbial biomass quantification in severely undernourished animals. In this context, hindgut-inhabiting fungi [39], which contribute a considerable percentage to the fecal microbial biomass, should not be neglected as often done [40]; this could be achieved by using advanced molecular genetic tools.

## 5. Conclusions

Reduction in feed intake of poor-quality roughage decreased fecal N, increased fungal C but did not affect bacterial C concentrations in feces of Boran cattle. Supplementation of a poor-quality roughage diet with sweet potato vine silage and urea molasses blocks did not affect the fecal chemical composition and fungal C but increased the bacterial C concentration in feces of Holstein × Boran heifers. Across all data, the fungal C/bacterial C ratio was positively related to N and negatively to NDF concentrations in feces, indicating diet-induced shifts in the fecal microbial community. Fungi contributed between 31 and 46% to the fecal microbial biomass and should not be neglected in the future analysis of the fecal microbiome, which is relevant for the fertilizer quality of cattle feces after application to soil.

## Figures and Tables

**Table 1:** Dry matter (DM), crude protein (CP), neutral detergent fiber (NDF), acid detergent fiber (ADF) and metabolizable energy (ME) concentrations of feedstuffs offered to cattle in feeding trials with steers (experiment 1) and heifers (experiment 2).

Trial	Feedstuff	DM	CP	NDF	ADF	ME
		(g kg^−1^ FM)	(g kg^−1^ DM)			(MJ kg^−1^ DM)
Experiment 1	Rhodes grass hay	916	33	769	494	6.3
	Cotton seed meal	924	297	508	362	8.3
	Molasses	699	26	ND	ND	10.8
Experiment 2	Roughage mix ^1^	778	71	735	470	ND
	Sweet potato vine silage	196	140	537	393	ND
	Urea-molasses block	899	374	27	17	ND

FM: fresh matter; ND not determined. ^1^ Roughage mix: 0.61 wheat straw + 0.39 Rhodes grass hay (on FM basis).

**Table 2:** Feed intake, diet digestibility and fecal concentrations of organic matter (OM), crude protein (CP) or N, neutral detergent fiber (NDF), and acid detergent fiber (ADF), as well as amino sugar components of fungal and bacterial biomass in feces of Boran steers fed diets corresponding to different levels (%) of maintenance energy requirements (MER); standard error of the mean (SEM); probability levels obtained with *lme* function.

Variable	Diet				Period				SEM	Probability Level		
	MER100	MER80	MER60	MER40	1	2	3	4		D	P	D × P
Feed intake (g kg^−0.75^ LW d^−1^)												
OM	74.4 a	58.6 b	51.8 c	36.9 d	60.4 a	53.4 bc	51.3 c	56.6 b	2.09	<0.01	<0.01	<0.01
CP	5.5 a	2.2 b	1.9 c	1.3 d	2.7 b	2.9 a	2.4 c	2.9 a	0.24	<0.01	<0.01	<0.05
NDF	56.0 a	49.2 b	43.5 c	31.0 d	48.4 a	43.9 bc	41.6 c	45.8 ab	1.45	<0.01	<0.01	<0.01
ADF	36.1 a	31.5 b	27.9 c	19.9 d	31.3 a	27.7 ab	27.4 b	29.1 ab	0.93	<0.01	<0.01	<0.01
Diet digestibility (g kg^−1^ DM)												
OM	591	601	590	574	607 a	571 b	577 b	601 a	4.6	NS	<0.01	NS
CP	492 a	203 b	133 bc	103 c	192 b	319 a	206 b	215 b	26.3	<0.01	<0.01	NS
NDF	562 b	608 a	597 a	581 ab	605 a	576 a	570 b	597 a	5.3	<0.01	<0.05	NS
ADF	512 c	568 a	549 ab	527 bc	559 a	514 b	532 ab	551 a	5.8	<0.01	<0.01	NS
Fecal composition (g kg^−1^ DM)												
OM	862 a	845 b	852 a	851 a	846 b	859 a	857 a	848 b	1.5	<0.01	<0.01	NS
N	12.8 a	10.3 b	10.4 b	10.2 b	11.0 ab	10.5 b	10.1 b	12.0 a	0.22	<0.01	<0.01	NS
NDF	695	696	703	702	684 c	703 ab	712 a	698 b	2.1	NS	<0.01	NS
ADF	500 ab	492 b	505 a	510 a	494 b	508 a	511 a	494 b	2.1	<0.01	<0.01	<0.05
Fecal microbial properties (mg g^−1^ DM)												
Muramic acid	0.62	0.62	0.67	0.65	0.54 a	0.55 a	0.74 b	0.74 b	0.013	NS	<0.01	<0.05
Galactosamine	1.8 b	3.0 a	2.3 b	2.2 b	2.0 ab	2.2 ab	1.8 b	2.3 a	0.05	<0.01	<0.01	<0.01
Glucosamine	2.6 b	2.7 b	3.0 a	2.8 ab	2.7 b	3.0 a	2.4 c	3.0 a	0.05	<0.01	<0.01	<0.01
Fungal C	12.1 b	13.1 b	15.1 a	13.4 b	14.2 a	13.9 a	11.6 b	14.0 a	0.27	<0.01	<0.01	<0.01
Bacterial C	28.1	27.8	30.4	29.2	24.3 b	33.1 a	24.8 b	33.3 a	0.60	NS	<0.01	<0.05
Microbial C	40.2 b	40.9 b	45.4 a	42.7 ab	38.5 b	47.0 a	36.4 b	47.3 a	0.73	<0.01	<0.001	<0.01
Fungal C/bacterial C	0.45 b	0.52 a	0.52 a	0.47 ab	0.61 a	0.43 b	0.49 b	0.43 b	0.012	<0.05	<0.001	<0.05

Periods: 1 = 25/07/2016–11/09/2016; 2 = 12/09/2016–30/10/2016; 3 = 31/10/2016–18/12/2016; 4 = 19/12/2016–23/01/2017; means within rows with different letters differ at *p* < 0.05 (Tukey’s test); NS = not significant.

**Table 3:** Feed intake, diet digestibility and fecal concentrations of organic matter (OM), crude protein (CP) or N, neutral detergent fiber (NDF), acid detergent fiber (ADF), and amino sugar components of fungal and bacterial biomass in feces of Holstein × Boran heifers offered roughage alone (R; wheat straw and Rhodes grass hay), or R supplemented with sweet potato vine silage (R + SPVS) and urea-molasses blocks (R + UMB); standard error of the mean (SEM); probability levels obtained with *lme* function.

Variable	Diet			Period		SEM	Probability Level		
	R	R + SPVS	R + UMB	1	2		D	P	D × P
Feed intake (g kg^−0.75^ LW d^−1^)									
OM	63.1	67.5	59.0	56.8	69.6	2.79	NS	NS	NS
CP	5.6	6.7	5.5	5.4	6.5	0.31	NS	NS	NS
NDF	50.4	52.6	47.7	44.5	55.9	2.22	NS	NS	NS
ADF	32.3	33.9	30.2	29.4	35.0	1.30	NS	NS	NS
Diet digestibility (g kg^−1^ DM)									
OM	509	539	512	515	525	7.8	NS	NS	<0.05
CP	337	385	325	356	342	16.7	NS	NS	NS
NDF	496	530	506	496	525	8.3	NS	NS	NS
ADF	428	458	429	427	449	6.8	NS	NS	NS
Fecal chemical composition (g kg^−1^ DM)									
OM	832 ab	836 a	829 b	819 b	846 a	4.2	<0.05	<0.01	NS
N	16.0	17.8	17.1	16.5	17.4	0.53	NS	NS	NS
NDF	679	662	679	667	680	3.9	NS	NS	NS
ADF	496	495	497	500	493	2.6	NS	NS	NS
Fecal microbial composition (mg g^−1^ DM)									
Muramic acid	0.42 b	0.53 a	0.52 a	0.47	0.51	0.024	<0.01	NS	NS
Galactosamine	1.3 b	1.5 a	1.6 a	1.3 b	1.6 a	0.06	<0.01	<0.05	<0.05
Glucosamine	2.5 a	2.9 a	2.9 a	2.6 b	3.0 a	0.10	<0.01	<0.05	NS
Fungal C	15.3	16.2	16.8	14.6 b	17.6 a	0.58	NS	<0.05	<0.01
Bacterial C	18.7 b	23.9 a	23.3 a	21.0	23.0	1.07	<0.01	NS	NS
Microbial C	34.0 b	40.1 a	40.1 a	35.6	40.6	1.47	<0.01	NS	NS
Fungal C/bacterial C	0.85 a	0.69 c	0.76 b	0.74	0.80	0.031	<0.01	NS	<0.01

Period 1: from 07/09/2015-11/10/2015; period 2: from 12/10/2015-15/11/2015; means within rows with different letters differ at *p* < 0.05 (Tukey’s test); NS: not significant.

**Table 4:** Fecal excretion (in g per 100 kg live weight; LW) of organic matter (OM), nitrogen, neutral detergent fiber (NDF) and acid detergent fiber (ADF) by Boran steers (experiment 1) fed diets corresponding to different levels (%) of maintenance energy requirements (MER) and by Holstein × Boran heifers (experiment 2) offered roughage alone (R; wheat straw and Rhodes grass hay), or R supplemented with sweet potato vine silage (R + SPVS) and urea-molasses blocks (R + UMB); standard error of the mean (SEM); probability levels obtained with lme function.

Diet	OM	N	NDF	ADF
	(g 100 kg^−1^ LW d^−1^)			
Exp. 1				
MER100	796 a	12 a	642 a	462 a
MER80	608 b	7 b	501 b	353 b
MER60	559 b	7 b	461 b	331 b
MER40	420 c	5 c	346 c	252 c
SEM	21.6	0.4	17.2	12.2
Probability level	<0.001	<0.001	<0.001	<0.001
Exp. 2				
R	884	17	721	527
R + SPVS	880	18	697	519
R + UMB	830	17	679	497
SEM	34.1	0.7	26.8	17.7
Probability level	NS	NS	NS	NS

Means within columns with different letters differ at *p* < 0.05 (Tukey’s test); NS = not significant.

**Table 5:** Pearson’s correlation coefficients and significance levels between fecal chemical composition and feed intake, ingesta composition and diet digestibility across experiment 1 and experiment 2 data sets.

Variable	N	NDF	ADF
	Fecal Chemical Composition (g kg^−1^ DM)		
Feed intake (g kg^−0.75^ LW)			
CP	0.79 ***	−0.49 ***	−0.26 *
NDF	0.42 ***	−0.29 *	−0.44 ***
ADF	0.41 **	−0.30 *	−0.42 ***
Diet digestibility (g kg^−1^ DM)			
CP	0.37 **	−0.26 *	NS
NDF	−0.52 ***	NS	NS
ADF	−0.59 ***	NS	NS

Statistical significance: * *p* ≤ 0.05, ** *p* ≤ 0.01, *** *p* ≤ 0.001, NS: not significant; LW: live weight, DM: dry matter, CP: crude protein, NDF: neutral detergent fiber, ADF: acid detergent fiber. Experiment 1: Boran steers fed at different levels (%) of maintenance energy requirements (MER) with Rhodes grass hay alone (MER40, MER60, MER80) or supplemented with concentrate (MER100); experiment 2: Holstein x Boran heifers fed roughage (R) alone or supplemented with sweet potato vine silage (R + SPVS) and urea molasses block (R + UMB).

**Table 6:** Pearson’s correlation coefficients between fecal concentrations of galactosamine, fungal C, bacterial and microbial C as well as intake, ingesta composition, diet digestibility and fecal composition, across experiment 1 and experiment 2 data sets.

Variable	Galactosamine	Fungal C	Bacterial C	Fungal C/
	(mg g^−1^ DM)			Bacterial C
Feed intake (g kg^−0.75^ LW)				
CP	−0.48 ***	NS	−0.26 *	0.34 **
NDF	NS	NS	NS	NS
ADF	−0.29 *	NS	NS	NS
Diet digestibility (g kg^−1^ DM)				
CP	−0.32 *	NS	NS	NS
NDF	0.46 ***	NS	0.27 *	−0.31 *
ADF	0.43 ***	NS	NS	−0.35 **
Fecal composition (g kg^−1^ DM)				
N	−0.45 ***	0.31*	−0.32 *	0.52 ***
NDF	0.41 **	NS	0.43 ***	−0.56 ***
ADF	NS	NS	NS	NS

Statistical significance: * *p* ≤ 0.05, ** *p* ≤ 0.01, *** *p* ≤ 0.001, NS: not significant; LW: live weight, DM: dry matter, CP: crude protein, NDF: neutral detergent fiber, ADF: acid detergent fiber. Experiment 1: Boran steers fed at different levels (%) of maintenance energy requirements (MER) with Rhodes grass hay alone (MER40, MER60, MER80) or supplemented with concentrate (MER100); experiment 2: Holstein x Boran heifers fed roughage (R) alone or supplemented with sweet potato vine silage (R + SPVS) and urea molasses block (R + UMB).

## Data Availability

Can be obtained for purposes of comparison or meta-analysis upon written.

## Supplementary Materials

The following are available online at https://www.mdpi.com/2076-2615/11/2/564/s1, Table S1: Non-significant influence of different sampling hours within a sampling day on faecal concentrations of amino sugars and microbial C (mg g^−1^ dry matter; DM) in trials of sub-maintenance feeding (Exp.1) and supplementation (Exp.2).
