# Supplementary material for: Feed Quality and Feeding Level Effects on Faecal Composition in East African Cattle Farming Systems
Source: Animals (Basel). 2021 Feb 22;11(2):564. doi: 10.3390/ani11020564 (PMC7927072; doi:10.3390/ani11020564)
Supplement: Supplementary file 1 [file animals-11-00564-s001.pdf]

Table S1. Non-significant influence of different sampling hours within a sampling day on faecal concentrations of amino sugars and microbial C (mg g^{-1} dry matter; DM) in trials of sub-maintenance feeding (Exp.1) and supplementation (Exp.2).

Faecal microbial properties (mg g^{-1} DM)	Hour 24:00	12:00	18:00	SEM	P value
Experiment 1					
Muramic acid	0.57	0.54	0.56	0.013	0.19
Galactosamine	2.0	2.0	2.0	0.06	0.45
Glucosamine	2.9	2.7	2.8	0.06	0.10
Fungal C	15.7	14.2	15.2	0.39	0.69
Bacterial C	25.6	24.3	25.3	0.61	0.19
Microbial C	41.3	38.5	40.5	0.85	0.21
Fungal C/bacterial C	0.65	0.61	0.63	0.019	0.26
Experiment 2					
Muramic acid	0.48	0.47	0.44	0.019	0.79
Galactosamine	1.3	1.3	1.3	0.05	0.42
Glucosamine	2.7	2.6	2.5	0.11	0.42
Fungal C	16.0	14.6	14.3	0.66	0.33
Bacterial C	21.5	21.0	19.9	0.88	0.53
Microbial C	37.5	35.6	34.1	1.39	0.53
Fungal C/bacterial C	0.76	0.74	0.74	0.029	0.83
